# Physical activity in physiotherapy students: Levels of physical activity and perceived benefits and barriers to exercise

**DOI:** 10.4102/sajp.v76i1.1399

**Published:** 2020-04-29

**Authors:** Diana Kgokong, Romy Parker

**Affiliations:** 1 Department of Physiotherapy, Faculty of Health Sciences, University of Cape Town, Cape Town, South Africa; 2 Pain Management Unit, Department of Anaesthesia and Perioperative Medicine, Neuroscience Institute, University of Cape Town, Cape Town, South Africa; 3 Groote Schuur Hospital, Cape Town, South Africa

**Keywords:** physical activity, benefits, barriers, physiotherapy, students

## Abstract

**Background:**

Physiotherapists have been identified as key role players in health promotion, prevention and treatment of non-communicable diseases.

**Objectives:**

The aim of this study is to describe the perceived benefits and barriers to exercise and their association with levels of physical activity (PA) in physiotherapy students attending university in the Western Cape province of South Africa.

**Method:**

This study follows a quantitative, cross-sectional, survey design. Two hundred and ninety-six participants were recruited from three universities in the Western Cape. Participants completed a demographic questionnaire (DQ), Exercise Benefits and Barriers Scale (EBBS) and the International Physical Activity Questionnaire (IPAQ).

**Results:**

Female students accounted for 83% of the sample. Out of the 296 participants, 58% lived off-campus and 65% were involved in sporting activities six hours per week. The median score on the EBBS was 136 (54–167) for all years. Responses with the highest agreement for perceived benefits were associated with physical performance. Alternatively, responses with the highest agreement for perceived barriers were associated with physical exertion. Only 37.5% students engaged in high PA.

**Conclusion:**

Undergraduate physiotherapy students in the Western Cape across all three universities do not engage in adequate PA. In this group of students, benefits associated with high PA related to physical performance and barriers associated with low levels of PA related to physical exertion.

**Clinical implications:**

Physiotherapists who do not practise what they preach are not effective role models and may not be effective in obtaining behaviour change through PA-related health promotion.

## Introduction

Physical activity (PA) cannot be separated from the practice of physiotherapy (Cup et al. 2007). For decades physiotherapists have been using PA and exercise to treat a range of conditions that include neuromuscular diseases, respiratory, orthopaedic, paediatric, non-communicable diseases (NCDs) and others (Cup et al. 2007; DeTurk & Scott 2008; Higgs, Refshauge & Ellis 2001; Meisingset et al. 2016). Today, NCDs have been noted to be the leading cause of death globally, reported to reach epidemic proportions and resulting in more deaths than all other causes combined (World Health Organisation 2009). Physical inactivity is the fourth leading risk factor for global mortality contributing to 6% of deaths globally (WHO 2018). Notably, one in four adults worldwide engages in insufficient PA (WHO 2017), and in South Africa, one in two adults engages in insufficient PA (Malambo et al. 2016). Furthermore, cardiovascular diseases are responsible for a third of deaths in the population globally; out of those deaths, 7.22 million are attributed to coronary heart disease (CHD) (Heran et al. 2011). Not only do NCDs contribute to mortality, but they also contribute to morbidity placing a burden of care on society. In South Africa, approximately 1.5 million people were diagnosed with diabetes in 2000; diabetes is the most common cause for non-traumatic amputations, a leading cause of blindness and is associated with end-stage kidney failure (Bradshaw et al. 2007). In addition to diabetes, South Africa has a high burden of cardiovascular diseases and obesity (Mayosi et al. 2009). Therefore, there is a high demand for physiotherapists to provide rehabilitative, preventive and education therapies, particularly in the prevention and management of NCDs (Bury & Moffat 2014; Dean 2009a, 2009b; Dean et al. 2011, 2014; Skinner 1980).

There is an ever-growing body of literature to support physiotherapists promoting the use of exercise and PA to successfully prevent and treat NCDs such as diabetes, cardiovascular disease, cancer, chronic lung disease, arthritis, liver disease, stroke, Alzheimer’s disease and others (Bury & Moffat 2014; Dean et al. 2016). Of course, the epidemiological literature has not only affected the practice of physiotherapy, but has also influenced the physiotherapy curriculum and the competencies that are needed to effectively address these 21st-century health challenges (Dean et al. 2011, 2016). However, the extent of the impact of evidence-based training on physiotherapy students’ health behaviours is a matter of speculation. For instance, although physiotherapy curricula worldwide emphasise the role of exercise and PA to optimise health, prevent illness and the use of exercise as a treatment technique, whether students are expected to engage in PA outside of the learning hours as part of their undergraduate training is unclear (Bodner et al. 2013). Theoretical knowledge about the benefits of PA and the methods of prescribing are fundamental during undergraduate training (Plotnikoff et al. 2015).

However, engaging in PA and exercise itself is beneficial for students to gain knowledge and facilitate learning while developing insights into the future challenges that they will face when implementing strategic health promotion in practice (Dabrowska-Galas et al. 2013; Shirley, Van der Ploeg & Bauman 2010).

One of the crucial components for success in achieving behaviour change for health promotion is for the prescriber to engage in the target behaviour, that is, PA (Dabrowska-Galas et al. 2013). The majority of patients will not start to engage in PA simply because it is advised (Keating et al. 2005). Achieving a change in behaviour requires the physiotherapist to engage with the patient’s beliefs and help in overcoming barriers to PA while also successfully modelling the behaviour change (Dean 2009b). Essentially, physiotherapists who lead by example are more likely to be successful in prescribing behaviour change (Dabrowska-Galas et al. 2013; Dean 2009a, 2009b; Dean et al. 2011, 2014, 2016).

Physiotherapists have been identified as key role players in health promotion, improving the health and well-being of communities by functioning both as role models and facilitators of behaviour change consistent with public health priorities (Chevan & Haskvitz 2010; Frerichs et al. 2012; Taukobong et al. 2014). As key role players engaged in PA promotion, it is valuable to investigate the health behaviours of physiotherapists and physiotherapy students alike.

There is a paucity of information on the level of PA of physiotherapists and physiotherapy students, making it difficult to predict their effectiveness as role models and the effectiveness of the health-promotion strategies they use.

This study was undertaken in South Africa, where physiotherapists complete a 4-year professional degree equivalent to an honours-level degree at university, undergoing training in theory and clinical practice at all levels of care (primary to quarternary). On completion of the degree, graduates are required to complete a 1-year community service placement and to register with the Health Professions Council of South Africa (HPCSA).

However, only upon completing the 1-year community service do they qualify to register as independent practitioners. Once registered as an independent physiotherapist, physiotherapists are licensed as direct access healthcare professionals. Given the key roles of exercise and PA as treatment tools in both the prevention and treatment of health conditions, and the value of physiotherapists being actively engaged in exercise to facilitate health behaviour change, this study aimed to describe the levels of PA and benefits and barriers to exercise for undergraduate physiotherapy students attending university in the Western Cape of South Africa.

## Methods

Our study followed a quantitative, cross-sectional, survey design. All male and female physiotherapy students over the age of 18 attending one of the three universities in the Western Cape Province of South Africa were recruited to the study. Students were completing their training in a 4-year BSc (Physiotherapy) programme.

No exclusion criteria applied. Each university had approximately 240 registered students, a total of 720 students in the population. Based on previous studies, using a population size of 720 (Kulavic et al. 2013) and a hypothesised 50% frequency of a ‘high number of barriers to participation’ and 5% confidence limits, a minimum sample size of 251 was required for 95% confidence.

Our study made use of three measurement tools. Firstly, a demographic questionnaire (DQ) was used to obtain the characteristics of the participants. To determine levels of PA, participants completed the short version of the International Physical Activity Questionnaire (IPAQ-short) (Dinger, Behrens & Han 2006). The IPAQ-short provides analysis algorithms for both the total volume and number of days to assess PA. The categorical score classifies PA into three levels: low, moderate and high levels of PA. Craig et al. (2003) undertook to determine the measurement properties (i.e. reliability and validity) of the IPAQ in 12 countries, including South Africa. To explore perceived benefits and barriers to exercise, the participants completed the 43-item Exercise Benefits and Barriers Scale (EBBS) questionnaire (Lovell, El Ansari & Parker 2010; Muzindutsi, Nishimwe-Niyimbanira & Sekhampu 2014; Sechrist, Walker & Pender 1987).

The EBBS is a 43-item questionnaire with a four-response, forced-choice Likert-type format with responses ranging from 4 (strongly agree) to 1 (strongly disagree) (Lovell et al. 2010; Muzindutsi et al. 2014; Sechrist et al. 1987). The scale is further divided into nine sub-groups, five sub-groups for perceived benefits and four sub-groups for perceived barriers, respectively (Sechrist et al. 1987). Scores on the instrument can range from 43 to 172; the lower the score, the more negatively the person perceives exercise and vice versa (Sechrist et al. 1987).

### Ethical consideration

Ethical approval was obtained for the Faculty of Health Sciences Research Ethics Committee of the University of Cape Town (HREC REF:712/2016). The heads of department of the three physiotherapy departments at the three universities in the Western Cape gave their permission for this study to be undertaken. Physiotherapy students at the three universities gave informed consent to participate in the study prior to the start of the study.

### Data analysis

The raw data were entered into Microsoft Excel. Data were labelled consistently and cleaned for missing values as well as non-plausible responses. The questionnaires included in this study provided ordinal and nominal data.

‘Statistica’ software (Hilbe 2007) was used for data analyses and the statistical significance value was accepted at *p* ≤ 0.05. Descriptive statistics were used to compare different categories of data such as males and females, universities and year of study. Non-parametric analysis was used because the results did not assume a normal distribution. Non-parametric measures of central tendency (i.e. median and range) are reported throughout.

Correlations were performed to explore associations between variables. Pearson’s chi-squared test was used to determine correlation in scores between students in different years because of the categorical nature of the data. Furthermore, the Kruskal–Wallis test was used to explore the association between scores on the EBBS and categories of PA (low, moderate and high).

## Results

Out of a population of 720 registered physiotherapy students from the three universities, 296 participated in the study (41%). The median age of the students was 22 years (18–29) (Table 1). The majority were female (83%), lived off-campus (58%) and were not working outside of their studies (73%).

**TABLE 1:** Characteristics of the participants (*n* = 296).

Demographics	Median	range	*n*	%
**Age**	22	18–29	-	-
Year 1 (*n* = 41)	18	18–22	-	-
Year 2 (*n* = 61)	19	18–23	-	-
Year 3 (*n* = 112)	21	19–27	-	-
Year 4 (*n* = 82)	22	19–29	-	-
**Gender**				
Female	-	-	246	83
Male	-	-	50	17
**Living on- or off-campus**				
On-campus	-	-	124	42
Off-campus	-	-	172	58
**Employment**				
Studying and employed	-	-	79	27
Studying only	-	-	217	73

### Levels of exercise and physical activity

**FIGURE 1:**
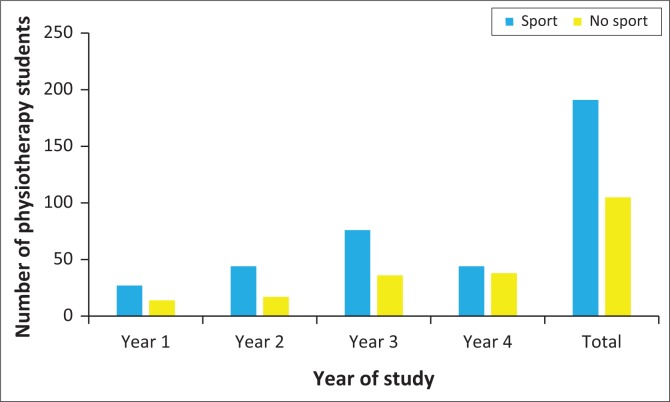
Number of physiotherapy students reporting participating in sporting activities on a weekly basis (*n* = 296).

The majority of students (196; 65%) reported participating in sporting activities, spending a median of 6 hours (4–8) per week doing sport (Figure 1).

**FIGURE 2:**
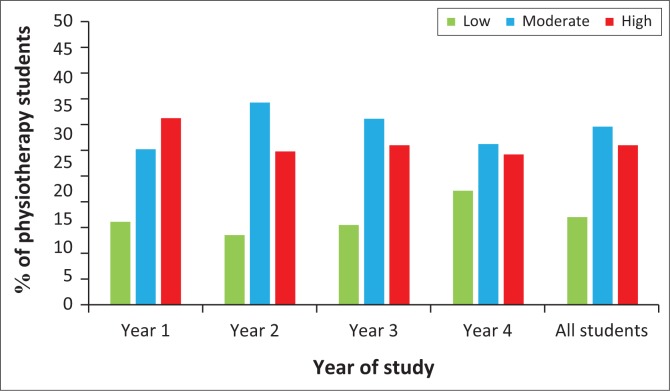
Percentage of students classified by level of physical activity based on year (*n* = 296).

The IPAQ-short scores revealed that overall the majority of students had low levels of PA. On the IPAQ, only 111 students (37.5%) reported high PA levels, while 61 students (20.6%) were classified as having low PA (Figure 2). There were no significant differences in levels of PA by year (*χ*^2^ = 4.02; *p* = 0.67).

**FIGURE 3:**
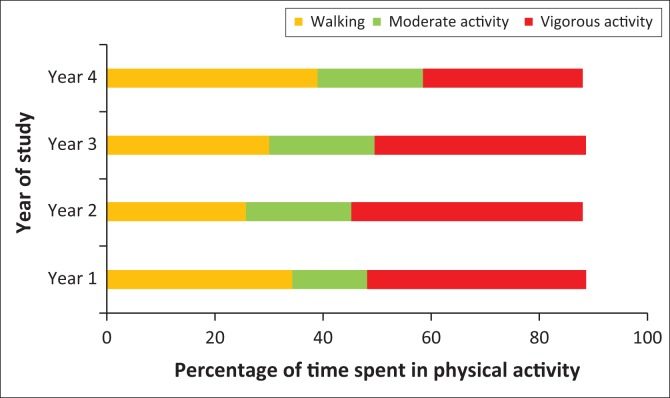
Percentage of time spent walking, doing moderate or vigorous physical activity by year (*n* = 296).

There were no differences between the proportion of students in different years of study and the percentage of time spent walking (*χ*^2^ = 4.55; *p* = 0.6), doing moderate PA (*χ*^2^ = 7.88; *p* = 0.98) or doing vigorous PA (*χ*^2^ = 9.66; *p* = 0.94) (Figure 3).

### Exercise benefits and barriers scale

The median score for all years on the Exercise Benefits and Barriers Scale was 136 (54–167); higher scores indicate more perceived benefits to exercise. In terms of perceived benefits from participating in PA, the participants had the highest agreement for the domains of ‘physical performance’ and ‘psychological outlook’ (Table 2). Participants either ‘agreed’ (score of 3) or ‘strongly agreed’ (score of 4) with most of the benefits under examination. For barriers to PA (Table 3), participants agreed most with items under the ‘physical exertion’ sub-scale and agreed the least with items under the ‘family discouragement’ sub-scale.

**TABLE 2:** The exercise benefits scale: Median and range of each questionnaire item.

Perceived benefit items	Median	Range
**Life enhancement sub-scale**		
25: My disposition is improved by exercise	3	1–4
26: Exercise helps me sleep better at night	3	1–4
29: Exercise helps me decrease fatigue	3	1–4
32: Exercising improves my self-concept	3	1–4
34: Exercising increases my mental alertness	3	1–4
35: Exercise allows me to carry out normal activities without becoming tired	3	1–4
36: Exercise improves the quality of my work	3	1–4
41: Exercise improves overall body functioning for me	3	2–4
**Physical performance sub-scale**		
7: Exercise increases my muscle strength	4	2–4
15: Exercise increases my level of physical fitness	4	1–4
17: Muscle tone is improved with exercise	4	1–4
18: Exercising improves functioning of my cardiovascular system	4	2–4
22: Exercise increases my stamina	4	2–4
23: Exercise improves my flexibility	3	1–4
31: My physical endurance is improved by exercising	4	2–4
43: Exercise improves the way my body looks	4	1–4
**Psychological outlook sub-scale**		
1: I enjoy exercise	3	1–4
2: Exercise decreases feelings of stress and tension for me	4	1–4
3: Exercise improves my mental health	4	2–4
8: Exercise gives me a sense of personal accomplishment	4	2–4
10: Exercising makes me feel relaxed	3	1–4
20: I have improved feelings of wellbeing from exercise	4	1–4
**Social interaction subscale**		
11: Exercising lets me have contact with friends and persons I enjoy	3	1–4
30: Exercising is a good way for me to meet new people	3	1–4
38: Exercise is good entertainment for me	3	1–4
39: Exercising increases my acceptance by others	2	1–4
**Preventative health sub-scale**		
5: I will prevent heart attacks by exercising	3	1–4
13: Exercising will keep me from having high blood pressure	3	1–4
27: I will live longer if I exercise	3	1–4

Likert scale responses: 1 = strongly disagree; 2 = disagree; 3 = agree; 4 = strongly agree.

**TABLE 3:** The exercise barriers scale: Median and range of each questionnaire item.

Perceived barriers items	Median	Range
**Exercise environment sub-scale**		
9: Places for me to exercise are too far away	3	1–4
12: I am too embarrassed to exercise	3	1–4
14: It costs too much money to exercise	3	1–4
16: Exercise facilities do not have convenient schedules for me	3	1–4
28: I think people in exercise clothes look funny	4	1–4
**42: There are too few places for me to exercise**	3	1–4
**Time expenditure sub-scale**		
4: Exercising takes too much of my time	3	1–4
24: Exercise takes too much time from family relationships	3	1–4
**37: Exercise takes too much time from my family responsibilities**	3	1–4
**Physical exertion sub-scale**		
6: Exercise tires me	2	1–4
19: I am fatigued by exercise	2	1–4
**40: Exercise is hard work for me**	2	1–4
**Family discouragement subscale**		
21: My spouse (or significant other) does not encourage exercising	4	1–4
**33: My family members do not encourage me to exercise**	4	1–4

Likert scale responses: 1 = strongly agree; 2 = agree; 3 = disagree; 4 = strongly disagree.

### Relationship between levels of physical activity and benefits and barriers to exercise

**FIGURE 4:**
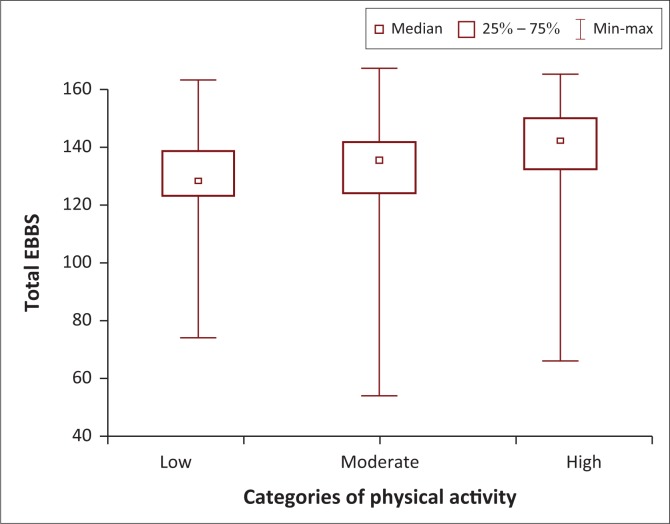
Box and whisker plot of scores on the Exercise Benefits and Barriers Scale for all students classified as undertaking low, moderate or high physical activity on the International Physical Activity Questionnaire-short (*n* = 295).

When we explored scores on the EBBS categorised by level of PA (low, moderate, high), we found that students who had high PA had significantly higher scores on the EBBS than those with moderate and low PA [*H* (2, *n* = 296) = 34.4 *p* < 0.01] (Figure 4). Students in their first year of study had no significant differences between the EBBS scores of students who had low, moderate and high PA levels [*H* (2, *N* = 41) = 3.01 *p* = 0.22]. However, students in the second (*n* = 61), third (*n* = 111) and fourth (*n* = 82) years of study with high PA levels had significantly better scores on the EBBS than those with low and moderate PA [*H* (2, *n* = 61) = 14.93; *p* < 0.001; *H* (2, *n* = 112) = 13.66; *p* = 0.001; *H* (2, *n* = 82) = 7.77; *p* = 0.02].

## Discussion

A total of 296 students, median age 22 years, participated in this study with 83% of the sample being female. They had a profile similar to that reported in studies involving physiotherapy students in Spain, Sri Lanka, Poland, the United Kingdom, Latvia and Australia (Dabrowska-Galas et al. 2013; Lovell et al. 2010; Mihailova et al. 2014; Plotnikoff et al. 2015; Poobalan et al. 2012; Ranasinghe et al. 2016; Shirley et al. 2010; Toloza et al. 2008). We found that 65% of the students participated in sporting activities, a median of 6 h per week. However, the IPAQ-short results are a reason for concern as the majority of students were either classified with ‘low’ or ‘moderate’ PA, that is, the students were not engaging in sufficient PA necessary for health benefits.

The IPAQ scores revealed that the majority of students across all years had low PA levels. Only 37.5% of the students engaged in high PA. The low percentage of students with high PA levels (37.5%) is similar to that reported in a study involving Spanish physiotherapy students (Toloza et al. 2008). According to the WHO, 41% of the population have low PA levels (Abubakari et al. 2009), similar to the 41.9% in our study. The PA behaviour of the physiotherapy students in our study appears to be no different from the PA behaviour of the general South African population (Steyn, Fourie & Temple 2006). Therefore, it is difficult to imagine how this group of students are going to model a positive health behaviour change.

These results are similar to previous research that reports a decline in PA levels in the years where young people undertake university studies (Mihailova et al. 2014; Plotnikoff et al. 2015). The low percentage of students with high PA levels in our study (37.5%) is similar to the 31.3% of Spanish physiotherapy students (Toloza et al. 2008). Although these results are disappointing and a reason for concern, the figures are more encouraging than the mere 15.9% of Sri Lankan physiotherapy students who presented with high PA levels (Ranasinghe et al. 2016). Given that 46% of Polish physiotherapy students reported high PA levels (Dabrowska-Galas et al. 2013), these results suggest that high PA levels may be related to culture and environment as well as knowledge of the benefits of PA.

The scores on the EBBS [136 (54–167)] suggest that a high number of the students have a positive view of exercise. These results are similar to those of general university students in both the United States (Grubbs & Carter 2002) and the United Kingdom (Lovell et al. 2010) for both perceived benefits and barriers. Despite the physiotherapy students’ anticipated additional knowledge and expertise in PA and exercise, physiotherapy students who participated in our study appear to have similar perceived benefits and barriers to exercise as non-physiotherapy students elsewhere.

Participants in the second (*n* = 61), third (*n* = 111) and fourth (*n* = 82) years of study with high PA had significantly better scores on the EBBS than those with moderate and low PA. These scores for participants with high PA may be a reflection of learning. First-year students may have the least knowledge and clinical practice of PA and positive health behaviour compared to those in the latter years. However, the trend of a reduction in levels of PA in year 4, despite perceived benefits, is concerning. An increase in knowledge may not be sufficient to offset other stressors that students face. Students in their fourth or final year of study in physiotherapy face higher academic pressures compared to other years (Ranasinghe et al. 2016). The academic pressure is reflected by students in one study where they reported being unable to participate in PA because of busy schedules (Ranasinghe et al. 2016). There is evidence that stress in physiotherapy students is on the rise; this stress is attributed to academic load, personality traits, illness and emotional problems among others (Davis et al. 2015).

Limitations of our study include the study design, data-collection methods, recruitment bias and limited generalisability. The cross-sectional survey design means that causality cannot be established. Thus, benefits and barriers to PA can only be studied as constructs. Secondly, data collection was conducted through self-report questionnaires, that is, IPAQ and EBBS. Although both the IPAQ and EBBS questionnaires have been previously validated and are reliable tools, self-report instruments are still vulnerable to bias. This potential for bias was most noticeable in the lack of agreement between the amount of PA reported by the students in the demographic questionnaire and the validated IPAQ. Thirdly, first-year students from one of the universities were unable to participate because of logistical issues, introducing a recruitment bias. Lastly, the results of our study are limited to universities in the Western Cape province of South Africa and cannot be generalised further.

Although our study has these limitations, one of the strengths of our study is the 95% confidence level that was achieved with a sample of *n* = 296. Therefore, these results can be generalised to physiotherapy students attending university in the Western Cape. Finally, to our knowledge, this is the first study in South Africa that describes the perceived benefits and barriers to exercise and their association with levels of PA in physiotherapy students.

Therefore, our study is breaking new ground for physiotherapists and their training in the South African context.

## Conclusion

Global mortality from NCDs is on the rise and physiotherapists need to respond by taking a leadership role. The levels of PA in physiotherapy students across South Africa need to be established. In addition, optimal PA-promotion strategies that take into account perceived benefits and barriers to exercise for this population need to be developed and tested. In conclusion, physiotherapy students should be trained in all the necessary competencies to be well equipped to handle the challenges of behaviour change in clinical practice as they journey towards being key role players by taking up a leading role in the fight against NCDs through PA-related health promotion. We propose that this training should include active engagement with both exercise and PA as part of the curriculum. Hence, future studies are indicated to explore the feasibility and effectiveness of including PA in the curriculum.

## References

[CIT0001] Abubakari A.R., Lauder W., Jones M.C., Kirk A., Agyemang C. & Bhopal R.S., 2009, ‘Prevalence and time trends in diabetes and physical inactivity among adult West African populations: The epidemic has arrived’, *Public Health* 123(9), 602–614. 10.1016/j.puhe.2009.07.009 19748643

[CIT0002] Bodner M.E., Rhodes R.E., Miller W.C. & Dean E., 2013, ‘Benchmarking curriculum content in entry-level health professional education with special reference to health promotion practice in physical therapy: A multi-institutional international study’, *Advances in Health Sciences Education* 18(4), 645–657. 10.1007/s10459-012-9404-x 22987193

[CIT0003] Bradshaw D., Norman R., Pieterse D. & Levitt N.S., 2007, ‘Estimating the burden of disease attributable to diabetes South Africa in 2000’, *South African Medical Journal* 97(8), 700–706. 17952227

[CIT0004] Bury T. & Moffat M., 2014, ‘Physiotherapists have a vital part to play in combatting the burden of noncommunicable diseases’, *Physiotherapy* 100(2), 94–96. 10.1016/j.physio.2014.03.004 24792243

[CIT0005] Chevan J. & Haskvitz E.M., 2010, ‘Do as I do: Exercise habits of physical therapists, physical therapist assistants, and student physical therapists’, *Physical Therapy* 90(5), 726–734. 10.2522/ptj.20090112 20299411

[CIT0006] Craig C.L., Marshall AL., Sjöström M., Bauman A.E., Booth M.L., Ainsworth B.E. et al., 2003, ‘International physical activity questionnaire: 12-country reliability and validity’, *Medicine & Science in Sports & Exercise* 35(8), 1381–1395. 10.1249/01.MSS.0000078924.61453.FB 12900694

[CIT0007] Cup E.H., Pieterse A.J., Jessica M., Munneke M., Van Engelen B.G., Hendricks H.T. et al., 2007, ‘Exercise therapy and other types of physical therapy for patients with neuromuscular diseases: A systematic review’, *Archives of Physical Medicine and Rehabilitation* 88(11), 1452–1464. 10.1016/j.apmr.2007.07.024 17964887

[CIT0008] Dabrowska-Galas M., Plinta R., Dabrowska J. & Skrzypulec-Plinta V., 2013, ‘Physical activity in students of the Medical University of Silesia in Poland’, *Physical Therapy* 93(3), 384–392. 10.2522/ptj.20120065 23086407

[CIT0009] Davis R., Campbell R., Hildon Z., Hobbs L. & Michie S., 2015, ‘Theories of behaviour and behaviour change across the social and behavioural sciences: A scoping review’, *Health Psychology Review* 9(3), 323–344. 10.1080/17437199.2014.941722 25104107 PMC4566873

[CIT0010] Dean E., 2009a, ‘Physical therapy in the 21st century (part I): Toward practice informed by epidemiology and the crisis of lifestyle conditions’, *Physiotherapy Theory and Practice* 25(5–6), 330–353. 10.1080/09593980802668027 19842862

[CIT0011] Dean E., 2009b, ‘Physical therapy in the 21st century (part II): Evidence-based practice within the context of evidence-informed practice’, *Physiotherapy Theory and Practice* 25(5–6), 354–368. 10.1080/09593980902813416 19842863

[CIT0012] Dean E., Al-Obaidi S., De Andrade A.D., Gosselink R., Umerah G., Al-Abdelwahab S. et al., 2011, ‘The first physical therapy summit on global health: Implications and recommendations for the 21st century’, *Physiotherapy Theory and Practice* 27(8), 531–547. 10.3109/09593985.2010.544052 21612551

[CIT0013] Dean E., Dornelas de Andrade A., O’Donoghue G., Skinner M., Umereh G., Beenen P. et al., 2014, ‘The second physical therapy summit on global health: Developing an action plan to promote health in daily practice and reduce the burden of non-communicable diseases’, *Physiotherapy Theory and Practice* 30(4), 261–275. 10.3109/09593985.2013.856977 24252072

[CIT0014] Dean E., Greig A., Murphy S., Roots R., Nembhard N., Rankin A. et al., 2016, ‘Raising the priority of lifestyle-related noncommunicable diseases in physical therapy curricula’, *Physical Therapy* 96(7), 940–948. 10.2522/ptj.20150141 26678448

[CIT0015] DeTurk W.E. & Scott L.B., 2008, ‘Physical therapists as providers of care: Exercise prescriptions and resultant outcomes in cardiac and pulmonary rehabilitation programs in New York State’, *Cardiopulmonary Physical Therapy Journal* 19(2), 35 10.1097/01823246-200819020-00002 20467497 PMC2845219

[CIT0016] Dinger M.K., Behrens T.K. & Han J.L., 2006, ‘Validity and reliability of the international physical activity questionnaire in college students’, *American Journal of Health Education* 37(6), 337–343. 10.1080/19325037.2006.10598924

[CIT0017] Frerichs W., Kaltenbacher E., Van de Leur J.P. & Dean E., 2012, ‘Can physical therapists Counsel patients with lifestyle-related health conditions effectively? A systematic review and implications’, *Physiotherapy Theory and Practice* 28(8), 571–587. 10.3109/09593985.2011.654179 22315947

[CIT0018] Grubbs L. & Carter J., 2002, ‘The relationship of perceived benefits and barriers to reported exercise behaviors in college undergraduates’, *Family & Community Health* 25(2), 76–84. 10.1097/00003727-200207000-00009 12010117

[CIT0019] Heran B.S., Chen J.M., Ebrahim S., Moxham T., Oldridge N., Rees K. et al., 2011, ‘Exercise-based cardiac rehabilitation for coronary heart disease’, *Cochrane Database of Systematic Reviews* (7), CD001800 10.1002/14651858.CD001800.pub2 21735386 PMC4229995

[CIT0020] Higgs J., Refshauge K. & Ellis E., 2001, ‘Portrait of the physiotherapy profession’, *Journal of Interprofessional Care* 15(1), 79–89. 10.1080/13561820020022891 11705073

[CIT0021] Hilbe J.M., 2007, ‘STATISTICA 7: An overview’, *The American Statistician* 61(1), 91–94. 10.1198/000313007X172998

[CIT0022] Keating X.D., Guan J., Piñero J.C. & Bridges D.M., 2005, ‘A meta-analysis of college students’ physical activity behaviors’, *Journal of American College Health* 54(2), 116–126. 10.3200/JACH.54.2.116-126 16255324

[CIT0023] Kulavic K., Hultquist C.N. & McLester J.R., 2013, ‘A comparison of motivational factors and barriers to physical activity among traditional versus nontraditional college students’, *Journal of American College Health* 61(2), 60–66. 10.1080/07448481.2012.753890 23409855

[CIT0024] Lovell G., El Ansari W. & Parker J.K., 2010, ‘Perceived exercise benefits and barriers of non-exercising female university students in the United Kingdom’, *International Journal of Environmental Research and Public Health* 7(3), 784–798. 10.3390/ijerph7030784 20617003 PMC2872307

[CIT0025] Malambo P., Kengne A.P., Lambert E.V., De Villiers A. & Puoane T., 2016, ‘Prevalence and socio-demographic correlates of physical activity levels among South African adults in Cape Town and Mount Frere communities in 2008–2009’, *Archives of Public Health* 74(1), 54 10.1186/s13690-016-0167-3 28042473 PMC5198503

[CIT0026] Mayosi B.M., Fisher A.J., Lalloo U.G., Sitas F., Tollman S.M. & Bradshaw D., 2009, ‘The burden of non-communicable diseases in South Africa’, *The Lancet* 374(9693), 934–947. 10.1016/S0140-6736(09)61087-4 19709736

[CIT0027] Meisingset I., Stensdotter A.K., Woodhouse A. & Vasseljen O., 2016, ‘Neck motion, motor control, pain and disability: A longitudinal study of associations in neck pain patients in physiotherapy treatment’, *Manual Therapy* 22, 94–100. 10.1016/j.math.2015.10.013 26586133

[CIT0028] Mihailova A., Kaminska I. & Bernane A., 2014, ‘Physical activity in physiotherapy and physical education high school students’, in SHS Web of Conferences, vol. 10, p. 00025, EDP Sciences, Daugavpils University, Daugavpils (Latvia), September 09, 2014.

[CIT0029] Muzindutsi P.F., Nishimwe-Niyimbanira R. & Sekhampu T.J., 2014, ‘Perceived benefits and barriers to physical exercise: A comparative analysis of first year and senior students at a South African university’, *African Journal for Physical Health Education, Recreation and Dance* 20(Suppl. 2), 169–181.

[CIT0030] Plotnikoff R.C., Costigan S.A., Williams R.L., Hutchesson M.J., Kennedy S.G., Robards S.L. et al., 2015, ‘Effectiveness of interventions targeting physical activity, nutrition and healthy weight for university and college students: A systematic review and meta-analysis’, *International Journal of Behavioral Nutrition and Physical Activity* 12(1), 45 10.1186/s12966-015-0203-7 25890337 PMC4393577

[CIT0031] Poobalan A.S., Aucott L.S., Clarke A. & Smith W.C.S., 2012, ‘Physical activity attitudes, intentions and behaviour among 18–25 year olds: A mixed method study’, *BMC Public Health* 12(1), 640 10.1186/1471-2458-12-640 22892291 PMC3490897

[CIT0032] Ranasinghe C., Sigera C., Ranasinghe P., Jayawardena R., Ranasinghe A.C., Hills A.P. et al., 2016, ‘Physical inactivity among physiotherapy undergraduates: Exploring the knowledge-practice gap’, *BMC Sports Science, Medicine and Rehabilitation* 8(1), 39 10.1186/s13102-016-0063-8 PMC5142393 27980791

[CIT0033] Sechrist K.R., Walker S.N. & Pender N.J., 1987, ‘Development and psychometric evaluation of the exercise benefits/barriers scale’, *Research in Nursing & Health* 10(6), 357–365. 10.1002/nur.4770100603 3423307

[CIT0034] Shirley D., Van der Ploeg H.P. & Bauman A.E., 2010, ‘Physical activity promotion in the physical therapy setting: Perspectives from practitioners and students’, *Physical Therapy* 90(9), 1311–1322. 10.2522/ptj.20090383 20651009

[CIT0035] Skinner M.A., 1980, ‘The influence of epidemics on the role of physiotherapists in rehabilitation’, *New Zealand Journal of Physiotherapy* 41(1), 22–25.

[CIT0036] Steyn K., Fourie J. & Temple N., 2006, *Chronic diseases of lifestyle in South Africa: 1995–2005*, pp. 33–47, South African Medical Research Council, Cape Town.

[CIT0037] Taukobong N.P., Myezwa H., Pengpid S. & Van Geertruyden J.P., 2014, ‘The degree to which physiotherapy literature includes physical activity as a component of health promotion in practice and entry level education: A scoping systematic review’, *Physiotherapy Theory and Practice* 30(1), 12–19. 10.3109/09593985.2013.783896 23808941

[CIT0038] Toloza S.C.M., Conesa A.G. & Montesinos M.D.H., 2008, ‘Prevalence of physical activity in physical therapy students of Murcia University’, *Fisioterapia-Barcelona* 30(4), 164–167. 10.1016/j.ft.2008.07.001

[CIT0039] World Health Organisation (WHO), 2009, *Global health risks: Mortality and burden of disease attributable to selected major risks*, World Health Organisation, Geneva.

[CIT0040] World Health Organisation (WHO), 2017, viewed 28 March 2017, from www.who.int/mediacentre/factssheets/fs385/en/.

[CIT0041] World Health Organisation (WHO), 2018, viewed 17 May 2017, from www.who.int/dietphysicalactivity/pa/en/.

